# Targeted Thyroid Screening in Patients With Vitiligo: A Practical Narrative Review

**DOI:** 10.7759/cureus.111952

**Published:** 2026-07-02

**Authors:** Sri Naidnur, Jesús Iván Martínez-Ortega

**Affiliations:** 1 Dermatology, Lake Erie College of Osteopathic Medicine, Greensburg, USA; 2 Histology, Autonomous University of Nuevo León, Monterrey, MEX; 3 Dermatology, Mexican Social Security Institute, Piedras Negras, MEX

**Keywords:** autoimmune, phenotype, review article, thyroid, vitiligo

## Abstract

Vitiligo is a chronic immune-mediated depigmenting disorder that is strongly associated with autoimmune thyroid disease, the most common autoimmune comorbidity reported in affected patients. Although thyroid abnormalities occur more frequently in patients with vitiligo than in the general population, recommendations regarding routine thyroid screening remain variable. Increasing evidence suggests that thyroid disease risk is not uniform across all patients with vitiligo and may be influenced by specific clinical and phenotypic characteristics.

This narrative review examines the epidemiology, temporal relationship, clinical predictors, and proposed pathogenic mechanisms linking vitiligo and thyroid disease, with a focus on practical screening considerations. Current evidence suggests that several clinical and phenotypic features are associated with an increased risk of thyroid dysfunction and autoimmune thyroid disease in patients with vitiligo, including nonsegmental vitiligo, acral involvement, female sex, extensive or progressive disease, longer disease duration, family history of thyroid disease or autoimmunity, the presence of additional autoimmune disorders, and symptoms suggestive of thyroid dysfunction. Thyroid autoantibody positivity is among the most frequently reported markers of thyroid autoimmunity, while subclinical hypothyroidism is among the most common forms of thyroid dysfunction in patients with vitiligo. Thyroid disease may precede or follow the onset of vitiligo, highlighting the importance of ongoing clinical awareness and individualized risk assessment.

A targeted, phenotype-guided approach to screening may improve diagnostic yield while reducing unnecessary testing in lower-risk individuals. Thyroid-stimulating hormone remains the most practical initial screening test, with additional laboratory evaluation guided by clinical presentation and individual risk factors. By integrating clinical phenotype, medical history, family history, symptoms, and physical examination findings into screening decisions, clinicians may be better positioned to identify patients most likely to benefit from thyroid evaluation and longitudinal follow-up while avoiding unnecessary testing. This practical approach may be particularly useful in primary care and dermatology settings.

## Introduction and background

Despite the well-established association between vitiligo and thyroid disease, approaches to thyroid screening remain variable in clinical practice. Routine laboratory screening for every patient with vitiligo remains debated because it may have low diagnostic yield in asymptomatic, lower-risk individuals, potentially resulting in unnecessary laboratory testing and the evaluation of clinically insignificant abnormalities; several studies have supported the consideration of a more targeted, risk-based approach to thyroid evaluation [1-3]. There is growing interest in identifying clinical features that may help recognize patients at higher risk for thyroid abnormalities. Factors such as nonsegmental vitiligo, the more common subtype that typically presents with bilateral, often symmetric depigmentation, unlike the usually unilateral and localized segmental subtype [4,5], acral involvement of the hands and wrists, female sex, extensive disease, and a family history of autoimmunity have all been associated with an increased risk of thyroid disease [1,4,5].

Vitiligo is a chronic depigmenting disorder characterized by immune-mediated loss of melanocytes and has an estimated global lifetime prevalence of approximately 0.36% [6]. It represents the most common cause of acquired depigmentation worldwide and carries a significant psychosocial burden [7]. Among the autoimmune conditions associated with vitiligo, autoimmune thyroid disease is the most consistently reported. This spectrum includes Hashimoto thyroiditis, an autoimmune condition that commonly leads to hypothyroidism, and Graves disease, which is characterized by autoimmune hyperthyroidism [8]. Thyroid dysfunction and thyroid autoantibody positivity are both reported more frequently in patients with vitiligo than in the general population [1,4,9]. Vitiligo is also associated with a broader spectrum of autoimmune comorbidities, of which thyroid disease carries the highest certainty of evidence [10].

The practical question is not whether vitiligo and thyroid disease are associated, but rather which patients with vitiligo should prompt thyroid testing. In this narrative review, we examine the clinical features associated with increased risk of thyroid disease and discuss a practical approach to targeted thyroid screening in everyday practice.

## Review

Search strategy and evidence synthesis

This narrative review was informed by a targeted literature search of PubMed, supplemented by manual review of the reference lists of relevant articles, through manuscript preparation in June 2026. Search terms included combinations of "vitiligo", "nonsegmental vitiligo", "segmental vitiligo", "phenotype", "thyroid disease", "autoimmune thyroid disease", "thyroid dysfunction", "Hashimoto thyroiditis", "Graves disease", "thyroid autoantibodies", "anti-thyroid peroxidase", "screening", and "guidelines". The literature was selected for relevance to the objectives of this review, with particular emphasis on systematic reviews, meta-analyses, clinical guidelines, expert consensus statements, and observational studies that evaluate thyroid disease risk, clinical predictors, phenotype-based risk stratification, and screening considerations in patients with vitiligo. The available evidence was narratively synthesized to provide a practical, clinically focused overview and to support the proposed phenotype-guided screening approach. All statistical values presented in this review, including prevalence estimates, odds ratios, confidence intervals, and p-values, were extracted from previously published studies. No original statistical analyses, pooled analyses, or meta-analyses were performed as part of this narrative review.

Epidemiology of the association

Autoimmune thyroid disease is generally considered the most common autoimmune comorbidity in patients with vitiligo, affecting approximately 15-25% of patients and occurring more frequently than alopecia areata, psoriasis, and many other autoimmune conditions reported in vitiligo cohorts [1,4,11]. Table 1 summarizes the common comorbidities associated with vitiligo.

**Table 1:** Common autoimmune and inflammatory comorbidities associated with vitiligo Approximate prevalence estimates were derived from published epidemiologic studies and systematic reviews of vitiligo and associated autoimmune comorbidities [1,4,9,11].

Comorbidity	Approximate prevalence among patients with vitiligo	Clinical implication
Autoimmune thyroid disease	~15-25%	The most important target for screening
Alopecia areata	~3-8%	Clinical examination and history
Psoriasis	~2-5%	Shared inflammatory pathways
Atopic dermatitis	~10-30%	Frequent coexistence

Multiple studies and meta-analyses have demonstrated a substantial association between vitiligo and thyroid disease. Fan et al. found that patients with vitiligo had approximately fourfold higher odds of thyroid disease (odds ratio (OR): 3.93; 95% confidence interval (CI): 2.23-6.93) and nearly sixfold higher odds of autoimmune thyroid disease (OR: 5.88; 95% CI: 2.68-12.89) compared with controls [4]. Similarly, Vrijman et al. reported pooled prevalences of thyroid disease, autoimmune thyroid disease, and thyroid-specific antibodies of 15.1%, 14.3%, and 20.8%, respectively, among patients with vitiligo [1].

Nonsegmental vitiligo, the most common subtype of vitiligo, appears to represent a higher-risk phenotype for thyroid disease and is more closely linked with systemic autoimmunity, thyroid dysfunction, thyroid autoantibody positivity, and other autoimmune conditions than segmental vitiligo [4,5].

Hashimoto thyroiditis, hypothyroidism, Graves disease, and subclinical thyroid dysfunction are among the most frequently reported thyroid abnormalities in patients with vitiligo [9]. Thyroid autoantibody positivity is among the most frequently reported abnormalities, with a prevalence approaching 20% [1]. Subclinical hypothyroidism is among the most common forms of thyroid dysfunction, with a reported prevalence of approximately 6% [9]. In contrast, overt hyperthyroidism has been reported in approximately 1-4% of patients and Graves disease in approximately 1-2% of cases [9]. Although less common than thyroid autoimmunity and hypothyroidism, these disorders remain more frequent than in the general population, where the prevalence of overt hyperthyroidism ranges from 0.2% to 1.4% [12]. Anti-thyroid peroxidase (anti-TPO) antibodies may also serve as an early marker of thyroid autoimmunity in selected patients [1,5]. Table 2 summarizes the spectrum of thyroid abnormalities reported in patients with vitiligo.

**Table 2:** Spectrum of thyroid abnormalities reported in patients with vitiligo Approximate prevalence estimates for thyroid abnormalities in patients with vitiligo were derived from published systematic reviews and meta-analyses [1,4,9,12].

Thyroid abnormality	Approximate prevalence in patients with vitiligo	Clinical significance
Thyroid autoantibody positivity	~20%	May precede overt thyroid dysfunction
Autoimmune thyroid disease	~14-25%	Most common autoimmune comorbidity
Thyroid disease (overall)	~15-25%	Supports targeted screening
Subclinical hypothyroidism	~6%	Often asymptomatic; may be detected only through screening
Overt hypothyroidism	Variable	Common manifestation of autoimmune thyroid disease
Overt hyperthyroidism	~1-4%	Less common but clinically significant
Graves disease	~1-2%	May present with ophthalmopathy and systemic symptoms

Several demographic and clinical features have been associated with increased thyroid disease risk, including female sex, longer disease duration, extensive or progressive disease, family history of thyroid disease or autoimmunity, and the presence of other autoimmune disorders [1,4,5,13]. Thyroid disease appears less common in childhood-onset vitiligo and more frequently reported in post-pubertal and adult-onset disease [4,5]. Table 3 summarizes the clinical patterns associated with increased risk of thyroid disease in patients with vitiligo.

**Table 3:** Higher-risk clinical patterns associated with thyroid disease in patients with vitiligo Clinical patterns associated with increased thyroid disease risk were synthesized from published observational studies and systematic reviews [1,4,5,13].

Higher-risk feature	Reported association
Nonsegmental vitiligo	Stronger association with autoimmune thyroid disease than segmental vitiligo
Acral involvement, especially hands and wrists	More frequently linked with thyroid abnormalities
Female sex	Higher prevalence of thyroid autoimmunity
Longer disease duration	Higher thyroid autoantibody positivity
Extensive or progressive disease	Higher autoimmune comorbidity burden
Family history of thyroid disease or autoimmunity	Increased autoimmune susceptibility
Additional autoimmune disease	Greater likelihood of thyroid dysfunction
Adult or post-pubertal onset	Thyroid disease is reported more commonly than childhood-onset vitiligo

Overall, epidemiologic data suggest that the risk of thyroid disease is concentrated in specific clinical subgroups, supporting a phenotype-guided approach to screening.

Pathogenic hypotheses (brief overview)

Vitiligo develops through immune-mediated destruction of melanocytes, leading to depigmented skin patches [7,8]. Autoimmune thyroid disease similarly results from immune-mediated injury to thyroid tissue, leading most commonly to Hashimoto thyroiditis or Graves disease [8].

The relationship between these conditions likely reflects shared autoimmune pathways, oxidative stress, and genetic susceptibility rather than two completely separate diseases [8,14,15].

Both melanogenesis and thyroid hormone synthesis originate from tyrosine-dependent oxidative biochemical pathways [8,16]. L-tyrosine serves as a common biochemical precursor for both melanin and thyroid hormone production, and melanocytes and thyrocytes generate reactive oxygen species, including hydrogen peroxide, as part of normal pigment and hormone synthesis [14,16]. Consequently, genetic or environmental factors that impair redox homeostasis may promote cellular injury, autoantigen exposure, and subsequent autoimmune responses directed against both tissues [14,16]. In genetically susceptible individuals, this oxidative stress-mediated toxicity has been proposed as a potential mechanism linking vitiligo and autoimmune thyroid disease [8,14,16].

Furthermore, genome-wide studies have identified susceptibility loci shared between generalized vitiligo and autoimmune thyroid disease, suggesting a heritable component to this co-occurrence that extends beyond coincidence [15].

Additional hypotheses suggest that structural similarities between thyroglobulin and melanocytic proteins such as tyrosinase and tyrosinase-related proteins (TYRP1/TYRP2) may contribute to autoimmune cross-reactivity [8]. In addition, thyroid-stimulating hormone receptor (TSH-R) expression has been demonstrated in keratinocytes and melanocytes, suggesting that thyroid-directed autoimmunity may influence cutaneous biology through mechanisms beyond systemic endocrine dysfunction [8,17]. Although these mechanisms remain incompletely understood, they provide a biologically plausible explanation for the frequent coexistence of vitiligo and autoimmune thyroid disease [8].

Although the exact mechanisms remain incompletely understood, this shared autoimmune background may help explain why thyroid abnormalities are more common in patients with vitiligo and supports a targeted, phenotype-guided approach to thyroid screening [1,8,18].

Temporal association and bidirectionality

Vitiligo and autoimmune thyroid disease appear to share a longitudinal and bidirectional relationship. Vitiligo may precede thyroid dysfunction by months or years, whereas autoimmune thyroid disease may already be present when vitiligo develops or may precede the onset of vitiligo in some patients [1,5,8].

Several studies provide additional insight into the timing of these conditions. In a cohort of 434 adults with nonsegmental vitiligo, 43 patients had previously diagnosed thyroid dysfunction. Among these patients, thyroid disease preceded vitiligo in 26 cases (60.5%), whereas vitiligo preceded thyroid disease in 13 cases (30.2%) [2,5]. Similarly, Lazzeri et al. found that autoimmune thyroid disease and thyroid nodules were significantly associated with adult-onset vitiligo and thyroid disease preceded vitiligo in a significant proportion of affected patients (p=0.014) [19]. Together, these findings support a bidirectional relationship and suggest that vitiligo and autoimmune thyroid disease may serve as clinical markers of a broader autoimmune predisposition rather than isolated organ-specific disorders. Thyroid abnormalities may remain subclinical for years before symptoms become apparent, making early recognition especially important [9].

The temporal variability of this relationship has important implications for screening. A patient with vitiligo may initially have normal thyroid studies and later develop thyroid dysfunction, whereas a patient with established autoimmune thyroid disease may subsequently develop vitiligo or another autoimmune disorder [2,5,19]. Therefore, a normal thyroid evaluation at the time of vitiligo diagnosis does not necessarily exclude future thyroid dysfunction. Taken together, these observations suggest that thyroid disease risk may evolve over time rather than remain fixed at diagnosis [1,5,8]. This supports ongoing clinical awareness and targeted follow-up in higher-risk patients rather than relying on a single screening assessment at diagnosis.

Clinical and phenotypic clues for suspicion (when to screen?)

Certain clinical and phenotypic features may help identify patients with vitiligo who are at higher risk for thyroid disease and may benefit from targeted thyroid screening rather than relying solely on universal laboratory testing.

One useful clinical clue described in the literature is acral involvement, particularly depigmentation affecting the hands and wrists. Multiple studies have suggested a stronger association between acral vitiligo and autoimmune thyroid disease and that acral involvement may represent a clinical phenotype associated with increased risk of thyroid disease [5].

This association is clinically important because thyroid abnormalities may occasionally be identified even in patients with limited cutaneous disease. Although isolated lesions alone should not automatically prompt laboratory evaluation in every patient, acral involvement may justify a lower threshold for thyroid screening, particularly when additional risk factors are present [5].

**Figure 1:**
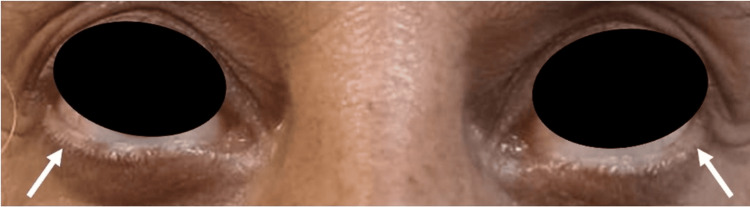
Subtle bilateral exophthalmos (white arrows) in a patient subsequently diagnosed with hyperthyroidism

**Figure 2:**
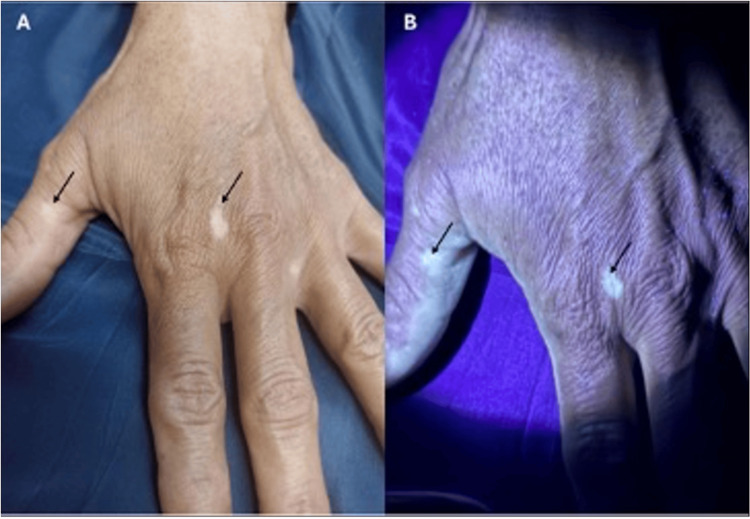
Localized acral nonsegmental vitiligo of the right hand (black arrows) (A) Clinical photograph showing depigmented lesions. (B) Wood's lamp examination demonstrating the accentuation of the lesions.

**Figure 3:**
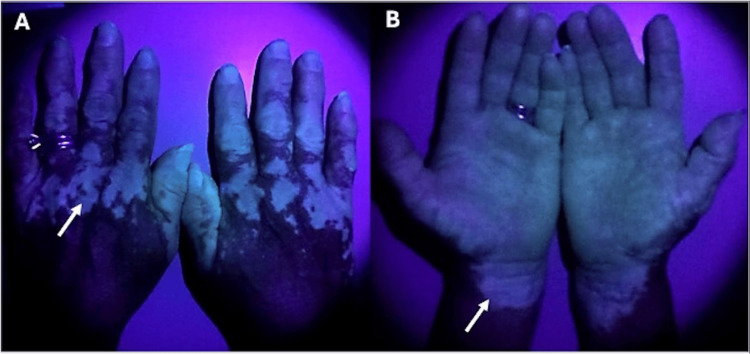
Wood's lamp examination demonstrating extensive acral nonsegmental vitiligo. Areas of depigmentation (white arrows) show bright accentuation under Wood's lamp illumination (A) Dorsal hands and fingers. (B) Palmar hands and wrists.

Figures 1-3 illustrate the clinical findings observed in patients evaluated in the authors' clinical practice and are included for educational purposes to highlight features relevant to thyroid screening in patients with vitiligo.

Nonsegmental vitiligo has been more consistently associated with autoimmune thyroid disease than segmental vitiligo [4,5]. Clinical history and physical examination may also provide important clues to underlying thyroid dysfunction. Findings such as goiter, tremor, tachycardia, ophthalmopathy, unexplained weight change, and heat or cold intolerance should prompt the consideration of thyroid evaluation [12,20].

Taken together, these findings support a practical and individualized approach to thyroid screening in patients with vitiligo. Rather than screening every patient in the same way, clinicians may consider the clinical phenotype, medical history, symptoms, family history, and physical examination findings when deciding whether thyroid evaluation is warranted [1,4,5].

Screening recommendations and practical approach (how to screen?)

Thyroid-stimulating hormone (TSH) is generally considered the most practical initial screening test in patients with vitiligo [3,21]. It is inexpensive, widely available, and sensitive for many forms of thyroid dysfunction [21]. If TSH is abnormal, additional evaluation with free thyroxine (free T4) and, when clinically indicated, triiodothyronine (T3) may help further characterize thyroid function [20,21].

Anti-TPO antibodies may be considered in selected higher-risk patients, particularly those with nonsegmental vitiligo, a family history of autoimmune thyroid disease, additional autoimmune disorders, or borderline thyroid function tests [1,3,9]. Because anti-TPO antibodies may be detected before overt thyroid dysfunction develops, they may help identify patients who warrant closer follow-up [1,9]. International expert recommendations support routine screening of anti-thyroid antibodies and thyroid function in patients with vitiligo [3].

Current evidence suggests that thyroid screening is most clinically useful in patients with the higher-risk clinical features summarized in Table 3, particularly when symptoms or signs of thyroid dysfunction are present [1,4,5]. Selected pediatric patients with nonsegmental disease, post-pubertal onset, symptoms, extensive disease, or a strong family history may also warrant consideration for screening [22-24].

Clinical judgment remains equally important. Symptoms such as fatigue, weight change, palpitations, tremor, heat or cold intolerance, constipation, menstrual irregularities, neck swelling, or ophthalmic findings should lower the threshold for thyroid evaluation regardless of vitiligo subtype or extent [12,20,21].

Conversely, routine laboratory screening may yield lower results in patients with stable, limited vitiligo who lack symptoms, concerning examination findings, a family history of thyroid disease, or other autoimmune risk factors [2,25]. In these patients, clinical observation and patient education regarding symptoms of thyroid dysfunction may be a reasonable approach.

A targeted screening strategy may improve diagnostic yield while reducing unnecessary testing, healthcare costs, and patient burden in lower-risk individuals [3,26]. Although formal cost-effectiveness studies are limited, a phenotype-guided approach may be a more practical and cost-effective strategy than routine laboratory evaluation for every patient with vitiligo [3,26].

Table 4 summarizes the clinical features that may help identify patients who are more likely to benefit from targeted thyroid screening.

**Table 4:** Evidence-informed, phenotype-guided thyroid screening considerations in patients with vitiligo TSH: thyroid-stimulating hormone; anti-TPO: anti-thyroid peroxidase Data synthesized from published systematic reviews, observational studies, clinical guidelines, and expert recommendations [1-5,9,12,20-27]. *Evidence levels were adapted from the Oxford Centre for Evidence-Based Medicine (OCEBM) 2009 framework: 1a: systematic review or meta-analysis; 2b: cohort study; 2c: cross-sectional or outcomes research; 3b: case-control study; 4: case series; and 5: expert opinion, narrative review, guideline recommendation, or clinical judgment [28].

Clinical feature	Why it matters/screening implication	Evidence level*
Symptoms suggestive of thyroid dysfunction	Symptoms such as fatigue, weight change, palpitations, heat or cold intolerance, constipation, dry skin, hair thinning, menstrual irregularities, eye symptoms, or neck swelling may indicate underlying thyroid disease; thyroid laboratory evaluation is reasonable	5
Nonsegmental vitiligo	More strongly associated with autoimmune thyroid disease than segmental vitiligo; baseline thyroid screening may be considered, particularly when additional risk factors are present	1a
Acral involvement, especially hands and wrists	Several studies have reported an association with thyroid abnormalities; a lower threshold for screening may be appropriate	2c
Female sex	Thyroid autoimmunity is reported more frequently in women with vitiligo; consider screening when additional risk factors are present	1a
Adult or post-pubertal onset	Thyroid disease appears more common in post-pubertal and adult patients; screening may be reasonable when additional risk factors are present	1a
Extensive or progressive disease	May reflect greater autoimmune burden and increased autoimmune comorbidity; consider thyroid screening and closer follow-up	2c
Longer disease duration	Associated with increased thyroid autoantibody positivity in some studies; repeat evaluation may be considered during follow-up	2c
Family history of thyroid disease or autoimmunity	Suggests increased autoimmune susceptibility; consider TSH ± anti-TPO antibodies	2c
Other autoimmune diseases	Supports broader autoimmune clustering; a lower threshold for thyroid evaluation may be appropriate	1a
Positive anti-TPO antibodies	May identify thyroid autoimmunity before overt thyroid dysfunction develops; periodic thyroid monitoring may be reasonable	1a
Pediatric patients with higher-risk features	Thyroid abnormalities may occur, particularly in post-pubertal patients or those with additional risk factors; consider selective screening	4
Stable, limited vitiligo without additional risk factors	Limited evidence supports routine laboratory screening in asymptomatic patients without clinical risk factors; clinical observation and symptom education may be sufficient	5

**Figure 4:**
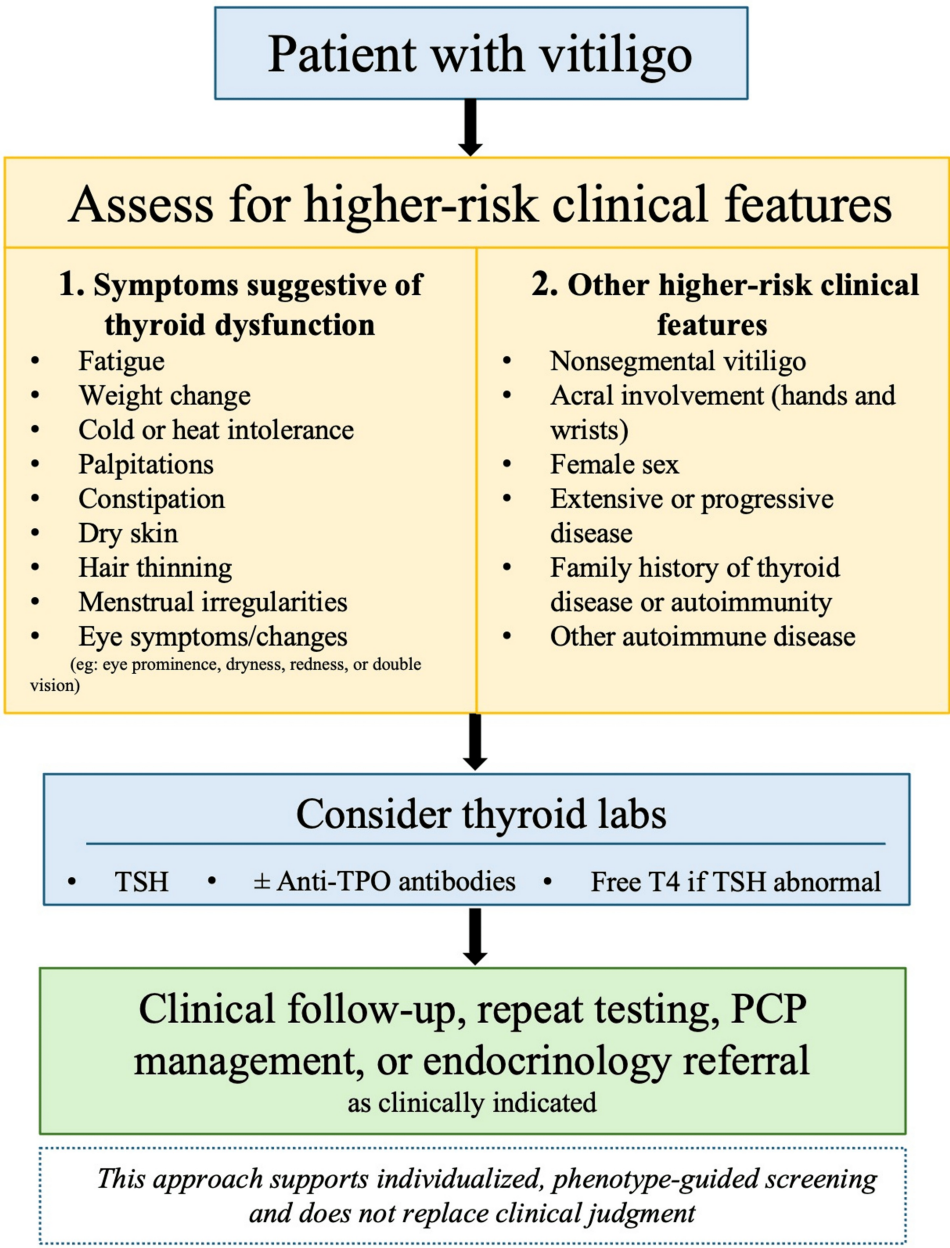
Practical, phenotype-guided thyroid screening approach in patients with vitiligo TSH: thyroid-stimulating hormone; anti-TPO: anti-thyroid peroxidase; PCP: primary care physician; free T4: free thyroxine This algorithm was developed by the authors using Microsoft PowerPoint (Microsoft Corporation, Redmond, Washington, United States) and is based on published studies and clinical guidelines [1,3-5,9,12,20,21,25,26]. It is intended to support clinical decision-making and does not replace clinical judgment or guideline recommendations.

A simplified phenotype-guided screening approach is presented in Figure 4.

## Conclusions

Vitiligo and autoimmune thyroid disease share a well-established clinical association, but thyroid disease risk is not uniform across all patients with vitiligo. A targeted, phenotype-guided approach to thyroid screening may help identify patients most likely to benefit from thyroid evaluation while avoiding unnecessary testing in lower-risk individuals. This practical approach may support individualized patient care in primary care and dermatology settings. Additional studies are needed to further define optimal screening strategies and long-term outcomes in patients with vitiligo.
